# Bacterial Community of Breast Milk in Breastfeeding Women Using Culture-Dependent and Culture-Independent Approaches

**DOI:** 10.4014/jmb.2407.07001

**Published:** 2024-09-09

**Authors:** Sumin Lee, Sojeong Heo, Mi-Kyung Park, Moon-Hee Sung, Do-Won Jeong

**Affiliations:** 1 Department of Food and Nutrition, Dongduk Women’s University, Seoul 02748, Republic of Korea; 2 School of Food Science and Biotechnology and Food and Bio-Industry Research Institute, Kyungpook National University, Daegu 41566, Republic of Korea; 3 KookminBio Corporation, Seoul 02826, Republic of Korea

**Keywords:** Breast milk, bacterial community, culture-dependent, culture-independent

## Abstract

This study aimed to analyze bacterial communities in breast milk obtained from five breastfeeding women. Culture-dependent and culture-independent methods were used to analyze microbial communities. Total bacterial count of breast milk determined using plate count agar ranged from 3.3 × 10^4^ ± 3.5 × 10^2^ colony forming unit (CFU)/g to 1.7 × 10^5^ ± 3.5 × 10^3^ CFU/g, with a pH between 6.4 and 6.8. Only three species, *Leuconostoc citreum* (17 out of 160 strains; 10.63%), *Staphylococcus epidermidis* (118 strains; 73.75%), and *Staphylococcus lugdunensis* (25 strains; 15.63%), belong to the phylum Bacillota were detected by culture-dependent analysis. Microbial communities analyzed via pyrosequencing revealed greater diversity compared to the culture-dependent analysis. At the phylum level, Bacillota accounted for 60.9% of the microbial community. At the genus level, *Staphylococcus* (24.57%), *Streptococcus* (22.93%), and *Methylobacterium* (8.76%) were dominant genera. While pyrosequencing demonstrated greater microbial diversity than the agar plate culture method, identified microbes might lack information or include many unculturable microbes. Most of all, considering the low total bacterial count averaging 7.2 × 10^4^ CFU/g, further research is needed to determine the significance of microbial presence in breast milk.

## Introduction

Breast milk is produced and secreted from alveoli in the breast under the influence of a hormone called prolactin, which is present in the blood. Breast tissue including alveoli has traditionally been considered sterile [1, 2]. However, with advancement of genetic analysis technologies such as pyrosequencing, it has been confirmed that microbes exist even in tissues previously considered aseptic. For example, bacteria have been reported to be present in breast milk [1, 3].

Breastfeeding is known to have a preventive effect on acute infectious diseases in infants [4], which might be attributed to the presence of lysozyme, antimicrobial substances, and antioxidants in breast milk. Such effect could be due to antimicrobial substances produced by beneficial microbes. Recent studies have reported that breast milk can inhibit the colonization of pathogenic microbes in newborns and facilitate the provision and colonization of probiotics [5]. Therefore, this study aimed to analyze microbial communities present in breast milk of breastfeeding women to determine the distribution of beneficial microbes. Furthermore, given differences observed in microbial communities between culture-dependent and culture-independent analysis methods in our previous experiments, we sought to apply both methods to confirm these differences. Results delineated characteristics of bacterial communities of breast milk from breast-feeding woman and suggested the involvement of diverse bacterial species.

## Materials and Methods

### Sample Preparation of Breast Milk

This study was approved by the Institutional Review Board of Dongduk Women's University (approval number: DDWU2206-04). Breast milk samples were collected from September 2022 to February 2023. Healthy breastfeeding mothers aged 20 to 40 years who had given birth between 36 and 40 weeks of gestation and had no history of being high-risk pregnant women within 6 months postpartum were chosen as subjects. Five breastfeeding women who breastfed infants under the age of one year were selected as participants (Table 1). Prior consent was obtained from all participating mothers. The collection procedure for samples involved mothers directly expressing breast milk into a breast milk storage bag (Bailey, Laon H&C CO., Ltd., Republic of Korea) until at least 50 ml was collected. The collected breast milk was stored frozen at -70°C and then aliquoted aseptically in 1 ml or 1 g portions for use during the study. Any remaining samples after the study's completion were discarded.

### pH and Total Bacterial Count

The pH measurement was conducted using a pH meter (ORION STAR A211, Thermo Fisher Scientific, USA). Total bacterial count was measured using Plate Count Agar (PCA; Becton, Dickinson and Co., USA) and observed after culturing at 30°C for 24 h.

### Identification of Culture-Dependent Isolates Using 16S rRNA Gene Sequence Analysis

Bacteria were isolated using Tryptic Soy Agar (TSA; Becton, Dickinson and Co.) and De Man, Rogosa, and Sharpe (MRS) agar (Becton, Dickinson and Co.) plates. Breast milk samples were diluted using 0.1% peptone water, followed by serial dilution in distilled water. They were then plated onto TSA and MRS agar. Plates were incubated at 30°C for 24 h. Over 20 colonies from each plate were collected based on differences in colony morphology and growth characteristics. Genomic DNAs from colonies were extracted using a DNeasy Blood & Tissue Kit (Qiagen, Germany). For amplification of the 16S rRNA gene, universal primers 27F (5'-AGA GTT TGA TCC TGG CTC AG-3') and 1492R (5'-GGT TAC CTT GTT ACG ACT T-3') [6] were used. PCR amplification was performed using a T3000 Thermocycler (Analytik Jena, Germany), with a mixture containing 10 ng of DNA, 0.5 mM of each primer, 1 U of Taq polymerase (Inclone Biotech Co., Republic of Korea), 10 mM dNTPs, and 2.5 mM MgCl_2_. PCR conditions were: initial denaturation at 95°C for 5 min, followed by 30 cycles of denaturation at 95°C for 1 min, annealing at 58°C for 1 min, and extension at 72°C for 1 min. PCR products were analyzed by Macrogen (Republic of Korea).

### Pyrosequencing for Culture-Independent Samples

To confirm bacterial communities using a culture-independent method, genomic DNA was extracted from breast milk using a DNeasy PowerSoil Kit (Qiagen). The quantity and quality of DNA were determined using PicoGreen and a Nanodrop. V3-V4 hypervariable regions of bacterial 16S rRNA genes from genomic DNA were amplified using a MyCycler Thermal Cycler (Bio-Rad, USA). Primers used for amplification were as follows: forward 5'- TCG TCG GCA GCG TCA GAT GTG TAT AAG AGA CAG CCT ACG GGN GGC WGC AG -3' and reverse 5'- GTC TCG TGG GCT CGG AGA TGT GTA TAA GAG ACA GGA CTA CHV GGG TAT CTA ATC C -3'. Each primer was linked to a 5' end-specific barcode sequence of 8 bases and a common linker sequence (TC for forward primer and CA for reverse primer).

Amplified fragments were subjected to pyrosequencing performed by Macrogen using a MiSeq platform (Illumina, USA). To evaluate richness estimators of bacterial species, diversity indices, and rarefaction curves, the Illumina sequencing pipeline of the Ribosomal RNA Database Project was applied [7]. PCR mixture consisted of 30 ng of genomic DNA, 50 pM of each primer, and Han-Taq polymerase (Genenmed, Republic of Korea). PCR was performed as follows: initial denaturation at 94°C for 90 s; 30 cycles of denaturation at 94°C for 45 s, annealing at 55°C for 45 s, and extension at 72°C for 45 s; and a final extension at 72°C for 5 min.

### Comparison between Culture-Dependent and Culture-Independent Analyses

Based on common species obtained from culture-dependent and culture-independent methods, a Venn diagram was presented based on InteractiVenn [8]. The diagram illustrates the number of species and their percentages.

### Statistical Analysis

Duncan’s multiple range test following a one-way analysis of variance (ANOVA) was used to evaluate significant differences between average values of pH, and log CFU. Values with *p* < 0.05 were considered statistically significant. All statistical analyses were performed using SPSS software v.27 (SPSS Inc., USA).

## Results and Discussion

### pH and Total Viable Counts of Breast Milk

Total bacterial counts of breast milk confirmed using PCA agar ranged from 3.3 × 10^4^ ± 3.5 × 10^2^ CFU/g to 1.7 × 10^5^ ± 3.5 × 10^3^ CFU/g, excluding sample M2 (Table 2). Previous studies on total bacterial counts of breast milk using PCA agar have shown a range of 1.2 to 5.5 log CFU/ml for South African mothers [9] and a range of 2 to 6 log CFU/ml for premature infants [10]. For breast milk in Switzerland, when using modified MRS agar, bacterial counts in the range of 2.1 to 2.3 log CFU/ml were detected [11]. Our study results of microbial total counts were also within this range. However, sample M2 did not show any detectable colonies on agar plates despite repeated attempts.

One of the factors affecting microbial growth is pH. To obtain basic information about the breast milk, its pH was confirmed to be between 6.4 and 6.8 (Table 2). A typical pH of breast milk is known to be between 6.5 and 7.7 [12]. Thus, pH values of samples used in this experiment also fell within this range.

### Bacterial Communities Identified Using Culture-Dependent Analysis

The microbial population in breast milk includes both aerobic and anaerobic bacteria. Additionally, among species detected through pyrosequencing, there are also anaerobic microorganisms such as *Cutibacterium acnes*, *Dakarella massiliensis*, *Polaromonas ginsengisoli*, and *Turicibacter bilis* [13-16]. Anaerobic microorganisms require longer cultivation time and more stringent culture conditions. This experiment aimed to not only compare methods for assessing microbial population in breast milk, but also isolate potential strains suitable for use as probiotics. Hence, both general anaerobic and aerobic culture media (MRS and TSA, respectively) were used to assess microbial population in breast milk. TSA is known to be suitable for general microbial isolation, while MRS is suitable for isolating lactic acid bacteria. When total bacterial count was measured using PCA agar, no microorganisms were detected in sample M2. Microorganisms were not detected using MRS or TSA either (Table 3). Excluding M2, a total of 160 strains were obtained after selecting 20 colonies from each breast milk sample on each medium. For M1 and M5, regardless of the type of medium used, all strains were identified as *Staphylococcus* (*S*.) *epidermidis*. In the case of M3, *Leuconostoc* (*Leu*.) *citreum* (17 strains) and *S. epidermidis* (3 strains) were detected on MRS agar, while *S. epidermidis* (19 strains) and *S. lugdunensis* (1 strain) were detected on TSA agar. For M4, regardless of the type of medium, *S. epidermidis* (8 strains per medium) and *S. lugdunensis* (12 strains per medium) were detected. Ultimately, excluding M2, breast milk samples yielded *Leu. citreum* (17 strains; 10.6%), *S. epidermidis* (118 strains; 73.8%), and *S. lugdunensis* (25 strains; 15.6%). In previous studies that used culture-dependent methods to analyze the microbial communities in breast milk, *S. epidermidis*, *Streptococcus* (*St.*), and *Bifidobacterium* (*B*.) *breve* were found to be dominant [17, 18]. Among these, *St. salivarius* and *B. breve* were isolated under anaerobic conditions [17, 18]. *S. epidermidis* is the most dominant under conditions similar to our experiment [17, 18]. Although culture-dependent methods can be influenced by the type of medium and isolation conditions, these results indicate that the *Staphylococcus* and *Streptococcus* genera are dominant.

### Bacterial Communities Identified Using Culture-Independent Analysis

To analyze bacterial communities from breast milk of five women, 124,858 - 226,024 sequences of sufficient quality were obtained (Table 4). The sequence coverage of these samples was 1.00, which provided sufficient statistical power to conduct analyses of bacterial communities except for M1. For M1, the Good's coverage was 0.97 and the Chao1 value was 3,882, indicating contamination rather than diversity of species. Therefore, results from this sample were excluded from analysis. Excluding M1, Chao1 and Shannon indices were analyzed for Amplicon Sequence Variant (ASV), revealing an average of 30-122 ASVs in breast milk samples. The Chao1 (species richness) and Shannon (species diversity) indices ranged from 30 to 122 (average, 76.5 ± 37.7) and from 2.80 to 5.45 (average, 4.48 ± 1.17), respectively. These results suggested that M1 was the richest in species and M2 was the most diverse. However, species diversity showed almost similar results among samples.

**Fig. 1:**
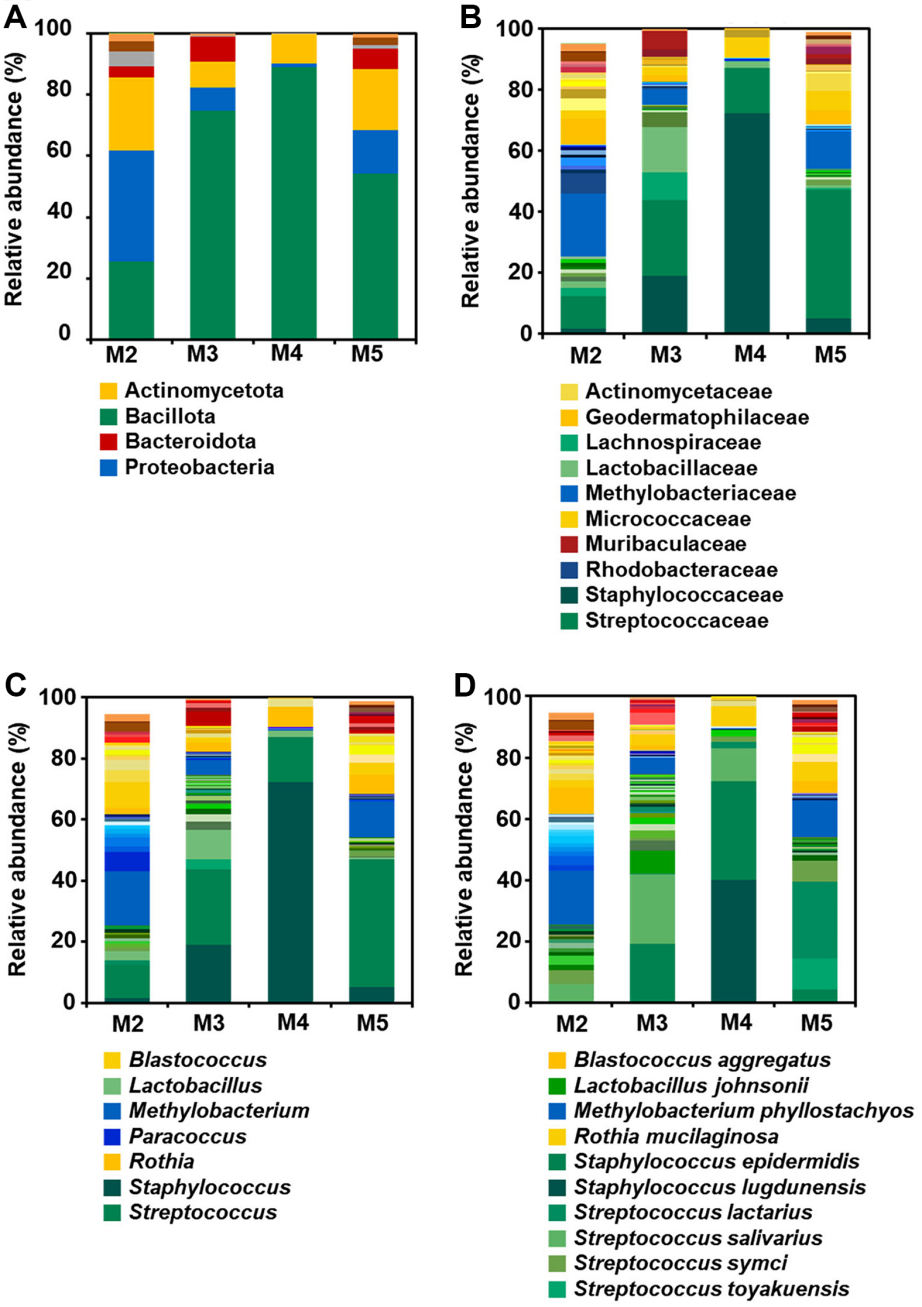
Bacterial species compositions of milk breast. Data portray phylum- (**A**) family- (**B**) genus- (**C**) and species- (**D**) levels of V3/V4 regions of 16S rRNA gene sequences. Regions > 200 bp were classified using the CD-HIT-OUT (97% confidence threshold). Categories of relative abundance > 5% are shown.

Except M1, the predominant phyla in the four breast milk samples were Bacillota (60.9%), Actinomycetota (15.4%), Proteobacteria (14.9%), and Bacteroidota (6.2%) (Fig. 1A). These four phyla accounted for 89.3% to 99.9% of the total microbiota composition, excluding M1. Microorganisms corresponding to Bacillota consisted of 3-13 families, 3-37 genera, and 7-48 species, while those belonging to Actinomycetota comprised 3-11 families, 3-12 genera, and 6-17 species. Proteobacteria encompassed microorganisms from 4-11 families, 4-13 genera, and 4-17 species, whereas Bacteroidota was not detected in M4.

Families of Staphylococcaceae (24.57%), Streptococcaceae (22.93%), Methylobacteriaceae (9.56%), and Actinomycetaceae (5.78%) were identified when breast milk samples were average values were checked (Fig. 1B). However, upon closer examination, slight differences were observed. In the case of M2, the order of these families in predominance was Methylobacteriaceae (20.56%) > Streptococcaceae (10.63%) > Geodermatophilaceae (8.61%) > Rhodobacteraceae (6.36%). For M3, it was Streptococcaceae (24.57%) > Staphylococcaceae (19.12%) > Lactobacillaceae (14.72%) > Lachnospiraceae (9.16%). For M4, it was Staphylococcaceae (72.31%) > Streptococcaceae (14.62%) > Micrococcaceae (6.54%) > Corynebacteriaceae (2.69%). For M5, it was Streptococcaceae (41.91%) > Methylobacteriaceae (11.97%) > Micrococcaceae (6.23%) > Actinomycetaceae (5.78%). Consequently, as indicated by average values, three families, Staphylococcaceae, Streptococcaceae, and Methylobacteriaceae, were predominant.

At the genus level, when examined closely, *Methylobacterium* (17.71%), *Streptococcus* (10.63%), and *Blastococcus* (8.61%) were the predominant genera for M2, while *Streptococcus* (24.57%), *Staphylococcus* (19.12%), and *Lactobacillus* (9.45%) were predominant for M3. In the case of M4, *Staphylococcus* (72.31%), *Streptococcus* (14.62%), and *Rothia* (6.54%) were predominant. For M5, *Streptococcus* (41.91%), *Methylobacterium* (11.97%), and *Rothia* (6.23%) were predominant (Fig. 1C). On average, *Staphylococcus* (24.57%), *Streptococcus* (22.93%), *Methylobacterium* (8.76%), and *Rothia* (4.69%) were predominant. To summarize the majority of results obtained so far, when the microbial communities in breast milk are analyzed using culture-independent methods, the genera *Staphylococcus* and *Streptococcus* are the most consistently and frequently observed [19-21]. This finding aligns with our results as well.

### Comparison of Bacterial Communities Identified Using Culture-Dependent and Culture-Independent Analyses

**Fig. 2:**
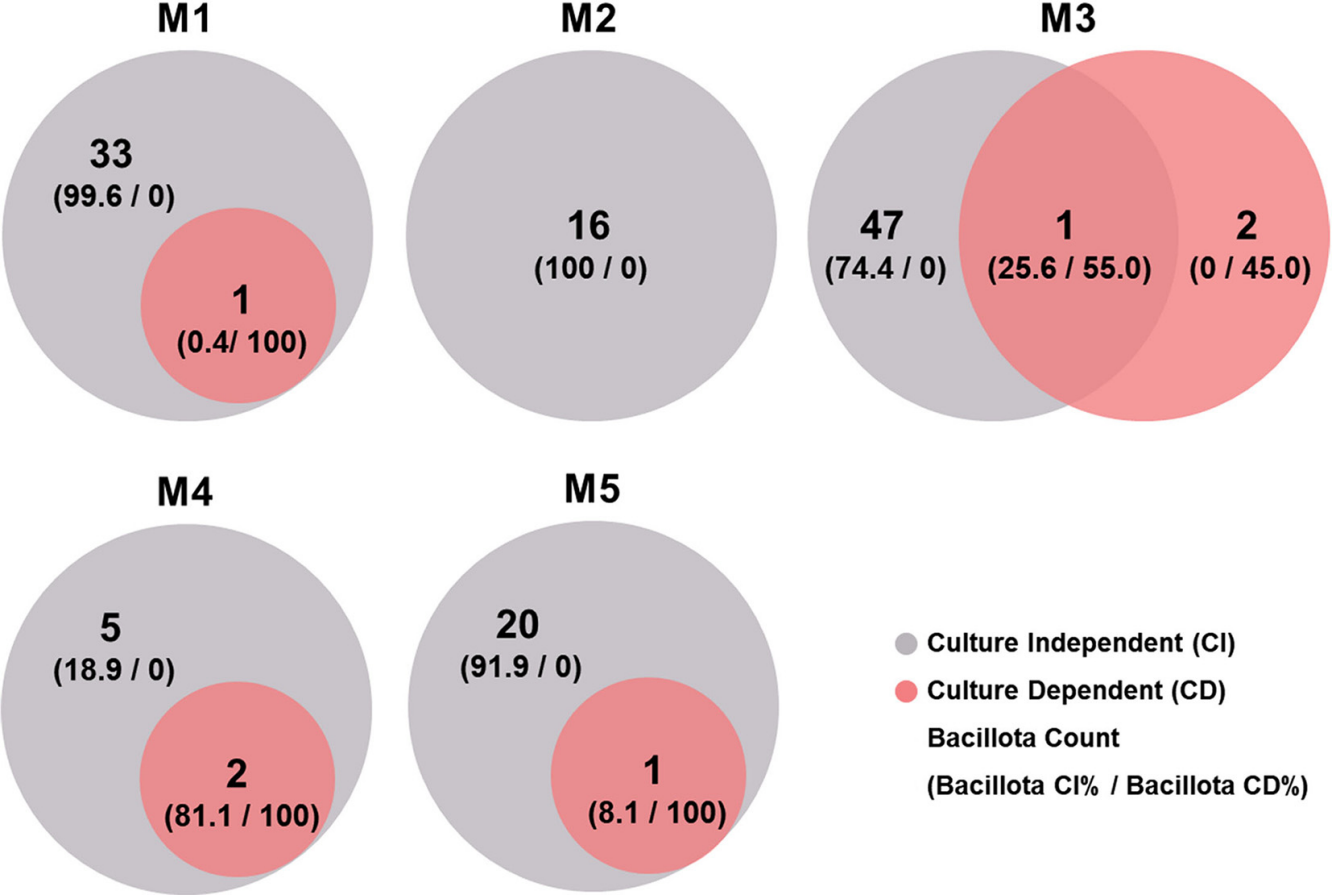
Proportional Venn diagram showing distribution of species in Bacillota phylum. Venn diagrams of results of culture-independent and -dependent analyses of five breast milk samples. Overlapping regions represent species shared between analytical methods, samples, or both. Numbers outside overlapping regions indicate numbers of species.

Results obtained through culture-independent analysis are often interpreted at the genus level rather than at the species level for accuracy. However, in our previous experiments, species-level results were also accurate [22-24]. Furthermore, since results obtained through culture-dependent analysis are at the species level (Table 3), we compared the two methods at the species level (Fig. 1D). As expected, culture-independent analysis showed greater diversity in bacterial communities than culture-dependent analysis (Table 3 and Fig. 1D). More specifically, microbial species obtained through culture-dependent analysis detected only three species, while culture-independent analysis detected a total of 248 species, showing a significant difference. Although a large difference was observed, we attempted to identify overlapping microbes in the same sample using a Venn diagram (Fig. 2). Microbes obtained through agar plates all belonged to the Bacillota phylum. Thus, we identified microbes corresponding to this phylum. In the case of M2, although a total of 16 species were detected through culture-independent analysis, no microbe was detected through culture-dependent analysis, resulting in no common microbe being found. For M3, 48 species were detected through culture-independent analysis, whereas only one species, *S. epidermidis*, of these 48 species was identified through culture-dependent analysis, leading to one common species (Fig. 2). For M4 and M5, seven and 21 species were detected through culture-independent analysis, respectively. Of them, only two and one species were identified from culture-dependent analysis, respectively, leading to two and one common species for M4 and M5, respectively.

No microbe was detected through culture-dependent method in the case of M2 as mentioned earlier. Therefore, it is possible that there is no overlapping species between microbes detected with culture-independent and culture-dependent methods. For M3, *S. epidermidis* was detected as a common species, accounting for 19.12% of microbes through a culture-independent method and 55% of microbes through a culture-dependent method. *Leu. citreum* and *S. lugdunensis* identified through a culture-dependent method were not detected through a culture-independent method. In the case of M4, *S. epidermidis* and *S. lugdunensis* were the two microbes confirmed through a culture-dependent method. They were also confirmed through a culture-independent method. These two species accounted for 72.31% of microbes identified through a culture-independent method. For M5, only one species, *S. epidermidis*, was confirmed through a culture-dependent method. It accounted for 4.38% of microbes identified through a culture-independent method.

Advantages and disadvantages of culture-dependent and culture-independent methods for analyzing microbial communities are widely reported. The most significant difference is that culture-dependent methods are limited to microbes that can be cultured, while culture-independent methods allow for analysis of microbial communities based on their DNA without biases of culture media. However, culture-independent methods also have limitations, such as incomplete detection of spore-forming microbes and inability to differentiate between viable and non-viable microbes. In this experiment, as observed, culture-independent methods detected a much larger number of microbial species than culture-dependent methods. Culture-based analysis only detected microbes belonging to the Bacillota phylum. Some species detected through culture-based methods were not detected by culture-independent methods. For example, *Leu. citreum* accounted for 42.30% of microbes isolated through culture-dependent methods in the M3 sample. However, it was not detected in any samples by culture-independent methods. Furthermore, *Leuconostoc* species were not detected with culture-independent methods. We found that sequences of V3-V4 region of 16S rRNA gene of *Leu. citreum* shared 75.7-83.4% similarities with species belonging to the Lactobacillaceae family, such as *Lactiplantibacillus pingfangensis*, *Lactobacillus crispatus*, *Lactobacillus johnsonii*, *Lactobacillus paragasseri*, and *Lactobacillus rogosae*, based on our analysis. This suggested that the discrepancy was not due to an error in culture-independent methods. However, in our recent research on kimchi added with seafood, *Leu. mesenteroides* was not well detected by culture-independent methods [25] possibly due to its physical characteristics such as cell wall structure, which might result in a lower recovery of DNA compared to other species. Therefore, although culture-dependent methods demonstrate microbial diversity, they are subject to biases in DNA recovery rate. Thus, further research is needed to understand reasons behind the dominance of *Leu. citreum* detected through culture-dependent methods but not through culture-independent methods.

Another notable observation was that *Streptococcus*, especially *St. salivarius*, which dominated in culture-independent analysis, was not detected in culture-dependent methods (Table 3 and Fig. 2). Detection rate of *St. salivarius* was 5.96% in M2, 22.79% in M3, and 10.64% in M4 by culture-independent analysis. However, it was not detected in this experiment, although it is a facultative anaerobic bacterium that grows well on MRS agar commonly used for culturing lactic acid bacteria. Cases like this, where microbes are detected by culture-dependent methods but not by culture-independent methods, are exemplified by spore-forming bacteria such as those in the *Bacillus* genus [22-24]. These bacteria are often found in the form of spores in the environment rather than as vegetative cells, making it difficult to recover DNA from thick spores with culture-independent methods, whereas they can grow from spores to vegetative cells on culture media, allowing their detection by culture-dependent methods. However, *Streptococcus* species are not spore-forming bacteria. Therefore, spore formation is not likely to be the reason for the discrepancy in detection between the two methods. Further research is needed to understand the reasons for differences in detection between the two methods.

Microbes such as *Staphylococcus* detected by culture-dependent methods are often found on human or animal skin [26, 27]. Additionally, *Leu. citreum* is commonly found in plants or dairy products [28, 29]. It can occasionally be found on surgical sites or in humans [30]. Therefore, it could be speculated that these strains might have been collected along with microbes from the skin during the process of collecting breast milk. This speculation is supported by the fact that Staphylococcaceae and Lactobacillaceae were not detected in M2, suggesting that hygiene status during the breast milk collection process might have influenced experimental results. Similarly, *St. salivarius* is known to primarily inhabit the oral cavity. It is possible that microbes transmitted from tongues of infants consuming breast milk could be collected during breastfeeding.

Finally, the total microbial count ranged from 10^5^ CFU/g to 10^6^ CFU/g, which represented the microbial count influenced by the bias of culture media. These microbial counts are very low compared to those found in fermented foods. In culture-dependent analysis (Table 3), only microbes suspected to originate from human skin or the oral cavity of infants were detected. Moreover, excluding these microbes, a total of 243 species, accounting for 9.02%, were detected by culture-independent analysis (Fig. 2). These results raise questions about whether these microbes truly exist in breast milk.

In conclusion, this study analyzed microbial compositions of breast milk using both culture-dependent and culture-independent methods, revealing clear differences between the two approaches. Microbes isolated through culture-dependent methods consisted of three species belonging to the Bacillota phylum: *S. epidermidis*, *S. lugdunensis*, and *Leu. citreum*. Among these, *S. epidermidis* and *S. lugdunensis* were also detected by culture-independent analysis. However, these two species are predominantly found on the skin of humans and animals, suggesting that they might have originated from the skin of mother during the breast milk collection process. Initially, we assumed that breast milk would contain numerous beneficial bacteria that could be transferred to infants. However, results of the experiment showed that the proportion of known beneficial bacteria was very low. Furthermore, considering the total microbial count and the microbial composition analyzed, doubts arose regarding the presence of microbes in breast milk. Therefore, further experiments are needed to resolve this question. We believe that results of this study will be helpful for interpreting the presence or absence of microbes in breast milk and for understanding its microbial compositions.

## Figures and Tables

**Table 1:** List of collected human breast milk.

Sample	Days after childbirth^a^	Collected date
M1	30	09.26.2022
M2	35	09.27.2022
M3	22	11.23.2022
M4	25	12.30.2022
M5	71	02.10.2023

^a^The time when the breast milk was collected, meaning the date after the newborn was born.

**Table 2:** The pH and bacteria cell counts of collected human breast milk.

No	pH	Cell count (CFU/g)
M1	6.5 ± 0.1 ^a^	3.3 × 10^4^ ± 3.5 × 10^2^ ^a^
M2	6.4 ± 0.1 ^a^	N.D
M3	6.4 ± 0.0 ^a^	3.8 × 10^4^ ± 3.9 × 10^3^ ^ab^
M4	6.5 ± 0.1 ^a^	1.7 × 10^5^ ± 3.5 × 10^3^ ^c^
M5	6.8 ± 0.1 ^b^	4.5 × 10^4^ ± 1.1 × 10^3^ ^b^

Bacteria were plated onto Plate Count Agar (PCA). Different letter indicates significant difference at *p* < 0.05 using Duncan’s multiple range test. CFU, colony forming unit; N,D., Non-detection.

**Table 3:** Numbers of bacterial species isolated from collected human breast milk.

Species	M1		M2		M3		M4		M5		Total
	MRS	TSA	MRS	TSA	MRS	TSA	MRS	TSA	MRS	TSA	
*Leuconostoc citreum*					17						17
*Staphylococcus epidermidis*	20	20			3	19	8	8	20	20	118
*Staphylococcus lugdunensis*						1	12	12			25
Total	20	20	0	0	20	20	20	20	20	20	160

MRS, *Lactobacilli* De Man, Rogosa and Sharpe Agar; TSA, Tryptic Soy Agar.

**Table 4:** Bacterial diversity indices of breast milk samples.

Sample name	High-quality reads	ASVs	Chao 1	Shannon	Gini-Simpson	Good’s coverage
M1	226,024	3,590	3,882.46	10.00	0.99	0.97
M2	143,032	81	81	5.45	0.95	1
M3	124,858	122	122	5.01	0.91	1
M4	191,086	30	30	2.80	0.76	1
M5	126,282	72	72	4.66	0.92	1
